# Optimizing surgical outcomes: preoperative embolization with graft placement in hypervascular, locally advanced high-grade differentiated thyroid carcinoma – a case report and review of the literature

**DOI:** 10.1097/RC9.0000000000001059

**Published:** 2026-09-29

**Authors:** Erwin Danil Yulian, Patrianef Darwis, Agnes Stephanie Harahap, Alvita Dewi Siswoyo, Faisal Mohammad Rifqi Aqil

**Affiliations:** a Oncology Division, Department of Surgery, RSUPN Dr. Cipto Mangunkusumo, Faculty of Medicine, Universitas Indonesia, Jakarta, Indonesia; b Vascular and Endovascular Surgery Division, Department of Surgery, RSUPN Dr. Cipto Mangunkusumo, Faculty of Medicine, Universitas Indonesia, Jakarta, Indonesia; c Department of Anatomical Pathology, RSUPN Dr. Cipto Mangunkusumo, Faculty of Medicine, Universitas Indonesia, Jakarta, Indonesia; d Department of Radiology, RSUPN Dr. Cipto Mangunkusumo, Faculty of Medicine, Universitas Indonesia, Jakarta, Indonesia; e Research Assistant in Division of Oncology Surgery, Department of Surgery, Faculty of Medicine, Universitas Indonesia – Cipto Mangunkusumo Hospital, Jakarta, Indonesia

**Keywords:** differentiated high-grade thyroid carcinoma, locally advanced, multidisciplinary approach, preoperative embolization, thyroid cancer treatment

## Abstract

**Introduction:**

A highly vascularized thyroid carcinoma presents a treatment challenge and may constitute a medical emergency. We report a rare case of preoperative embolization for the treatment of profuse bleeding from thyroid carcinoma and review the relevant literature.

**Case presentation:**

A 52-year-old male presented with a bleeding neck mass, pain, and dysphagia. The mass enlarged within 1 year to 15 × 13 × 10 cm with prominent ulceration, necrosis, pus, and bleeding. A CT scan showed a solid mass in the right neck originating from the right lobe and isthmus of the thyroid, compressing the larynx, trachea, hypopharynx, and esophagus, and displacing the right sternocleidomastoid and strap muscles. CT angiography showed a hypervascular tumor with multiple branches originating mainly from the right external carotid artery. Bleeding subsided after multiple vascular interventions consisting of embolization and placement of a BeGraft stent between the right common carotid artery and right internal carotid artery. The patient subsequently underwent debulking followed by total thyroidectomy with a double deltopectoral flap. Immunohistochemistry confirmed differentiated high-grade thyroid carcinoma.

**Discussion:**

Management of locally advanced thyroid cancer requires a multidisciplinary approach, emphasizing complete resection while preserving vital structures. In this case, significant vascular involvement necessitated resection of the internal jugular vein and preoperative embolization, which reduced intraoperative blood loss. Although rare, preoperative embolization appears to be safe and beneficial in selected cases of thyroid cancer.

**Conclusion:**

This case underlines the importance of a personalized multidisciplinary approach in the treatment of highly aggressive thyroid cancer. A review of similar studies shows that preoperative embolization is safe and effective in the initial treatment of highly vascularized thyroid carcinoma.

## Introduction

Differentiated high-grade thyroid carcinoma (DHGTC) was recently recognized within the spectrum of high-grade, non-anaplastic, follicular cell–derived carcinomas, as defined in the World Health Organization 2022 classification of Endocrine and Neuroendocrine Tumors^[^1^]^. DHGTC typically presents as a large (>4 cm), highly vascularized tumor with infiltration into surrounding structures, including vascular structures, perithyroidal skeletal muscle, neural structures, and surrounding lymph nodes^[^2,3^]^. High-grade non-anaplastic carcinomas of follicular cells are rare, ranging from 1% to 6.7% of all thyroid carcinomas, but account for a high proportion of thyroid cancer-related deaths^[^4^]^.

HIGHLIGHTS

- Embolization and graft placement can stop bleeding in hypervascular tumors.
- Multidisciplinary collaboration is essential for treating locally advanced thyroid cancer.
- Treatment and prognosis for high-grade thyroid carcinoma lie between those of differentiated thyroid carcinoma and advanced thyroid cancer.

At present, standardized treatments for DHGTC have not been clearly established, and management is often individualized on a case-by-case basis, incorporating surgery with selected adjuvant therapies. The available literature on this topic is still limited, mostly because this is a new classification, but current evidence suggests that the prognosis of DHGTC lies between well-differentiated tumors, such as follicular thyroid carcinoma, papillary thyroid carcinoma (PTC), and oncocytic carcinoma of the thyroid (OCA), and anaplastic thyroid carcinoma^[^5^]^.

The aggressive and hypervascular nature of DHGTC predisposes patients to significant complications, including severe hemorrhage from major thyroid vessels, which may present as a life-threatening emergency. In this article, we present a complicated case of locally advanced DHGTC with active bleeding and infiltration into surrounding vascular structures. Given the rarity of vascular-based interventions in the management of thyroid carcinoma, we also provide a focused review of the existing literature on the use of preoperative embolization and endovascular techniques in similar clinical contexts.

## Case presentation

A 52-year-old male presented with an enlarging right-sided neck mass, initially the size of a quail egg, with pain and difficulty swallowing that had persisted for over a year. The patient first noticed the mass in 2020 but did not seek medical help at that time and relied solely on herbal remedies. The mass progressively enlarged and became painful over the following years, eventually ulcerating in July 2023. The 3-year interval between symptom onset and diagnostic confirmation, attributable to delayed health-seeking behavior and reliance on herbal treatment, likely contributed to the advanced local extent of the disease at presentation. He had a 30-year smoking history and had received herbal treatment, but the mass continued to enlarge and eventually ruptured. Clinical examination revealed a large anterior neck mass measuring 15 × 13 × 10 cm, with ulceration, necrosis, pus, tenderness, and continuous bleeding (Fig. 1). The patient developed severe anemia due to chronic bleeding, with a hemoglobin level falling to less than 4 g/dL, requiring repeated transfusions. Hemostatic measures and epinephrine failed to control the bleeding.

**Figure 1.**
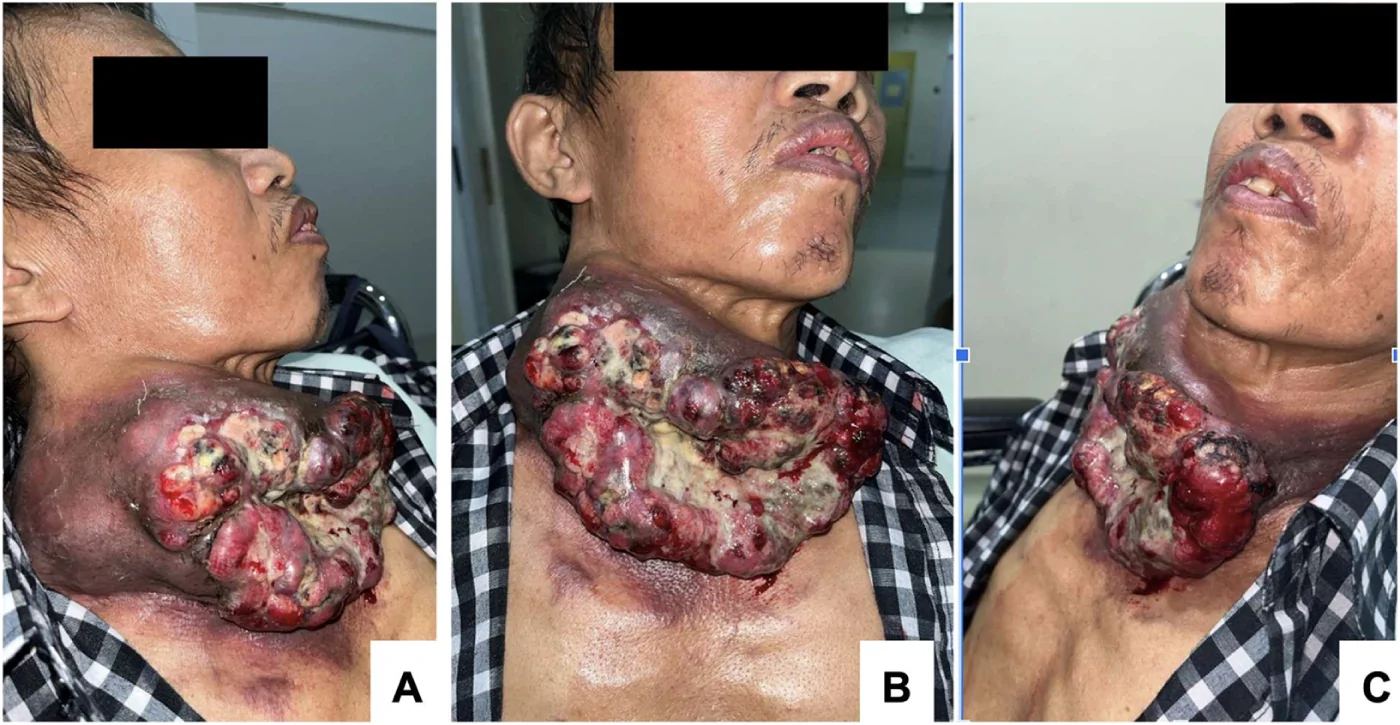
Clinical presentation of bleeding neck mass: (A) right side; (B) front side; (C) left side.

Laboratory findings showed normal thyroid hormones (TSH: 4.8; thyroglobulin (Tg): 21.96). Core needle biopsy revealed a Category IVc Hürthle cell neoplasm, according to the Korean Thyroid Association. Neck CT revealed a solid mass originating from the right lobe and thyroid isthmus, displacing the larynx, trachea, hypopharynx, and esophagus without airway narrowing, which was classified as SHIN Classification grade II (SHIN II). Anteriorly, the mass infiltrated the right sternocleidomastoid and strap muscles, causing cutis-subcutis ulceration (Fig. 2 A I). CT angiography demonstrated multiple arterial feeders and encasement of the right carotid artery and internal jugular vein (IJV), with collateral venous circulation (Fig. 2 A II–VI).

**Figure 2.**
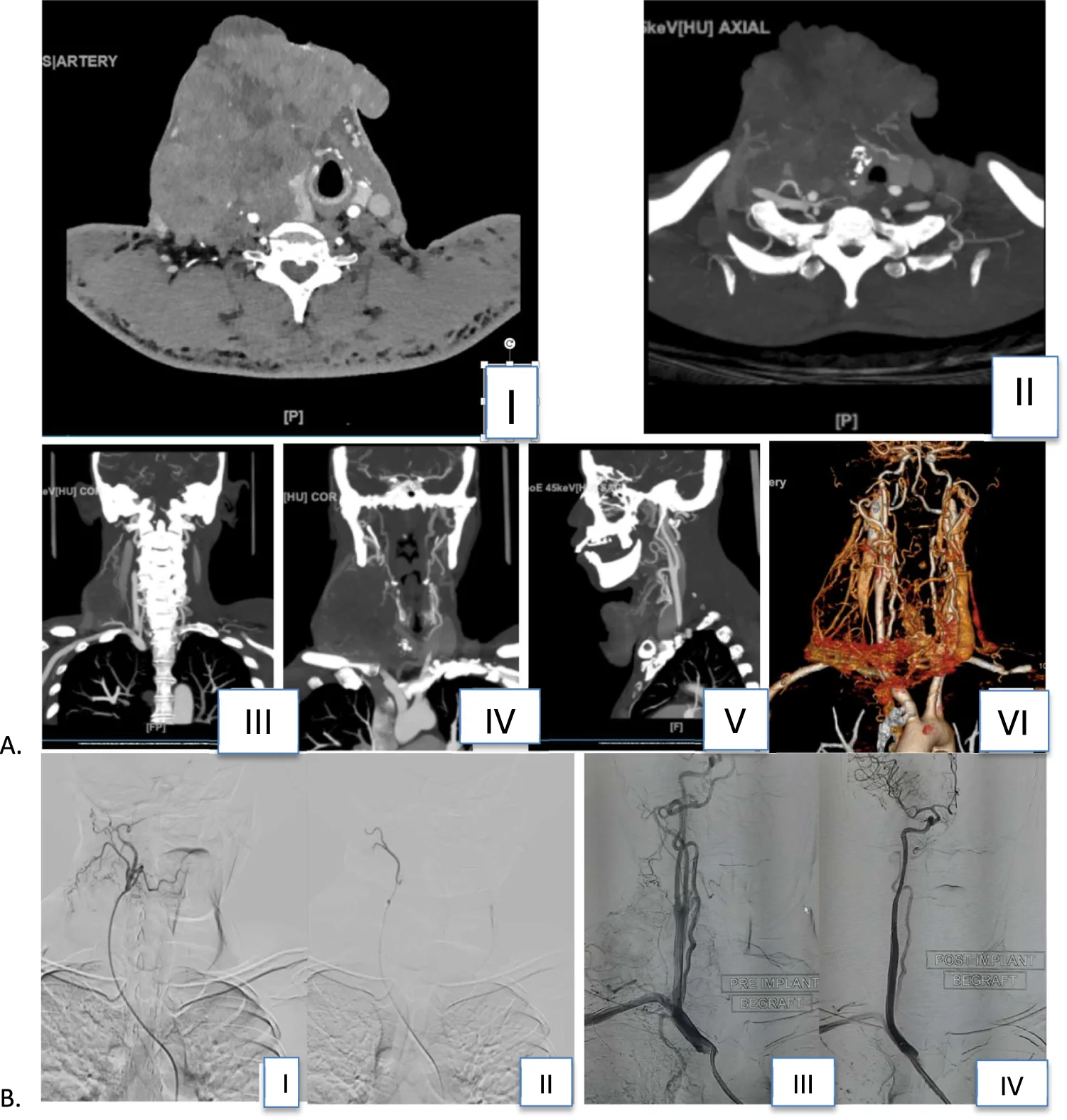
(A) CT angiography showing hypervascularity of the mass; (B) first embolization of the feeding artery from the right external carotid artery with PVA; (B I) before embolization; (B II) after embolization; placement of a BeGraft between the right common carotid artery and the right internal carotid artery; (B III) preembolization; (B IV) postembolization.

The bleeding was considered an emergency and managed with multiple vascular interventions. The first embolization targeted multiple feeding arteries of the thyroid tumor originating from the right external carotid artery, using polyvinyl alcohol particles (350–500 μm). The patient’s condition improved, with a brief episode of fever. Persistent bleeding necessitated a second intervention 3 weeks later, involving placement of a 6 × 58 mm balloon-expandable covered stent (BeGraft stent; Bentley InnoMed GmbH, Hechingen, Germany) at the common–internal carotid junction (Fig. 2 B).

The following day, debulking and total thyroidectomy with reconstruction using double deltopectoral flaps and an infraumbilical full-thickness skin graft were performed. The tumor was dissected from the platysma to the sternocleidomastoid muscle. The strap muscles and right IJV were resected due to infiltration, with preservation of the recurrent laryngeal nerve (RLN) and shaving of the trachea. A level IV left lymph node (0.9 × 0.7 × 0.6 cm) was also excised. Estimated intraoperative blood loss was approximately 2000 mL. Hemostasis was secured, the wound was irrigated, two vacuum drains were placed, and clips were applied to the residual tumor (Fig. 3).

**Figure 3.**
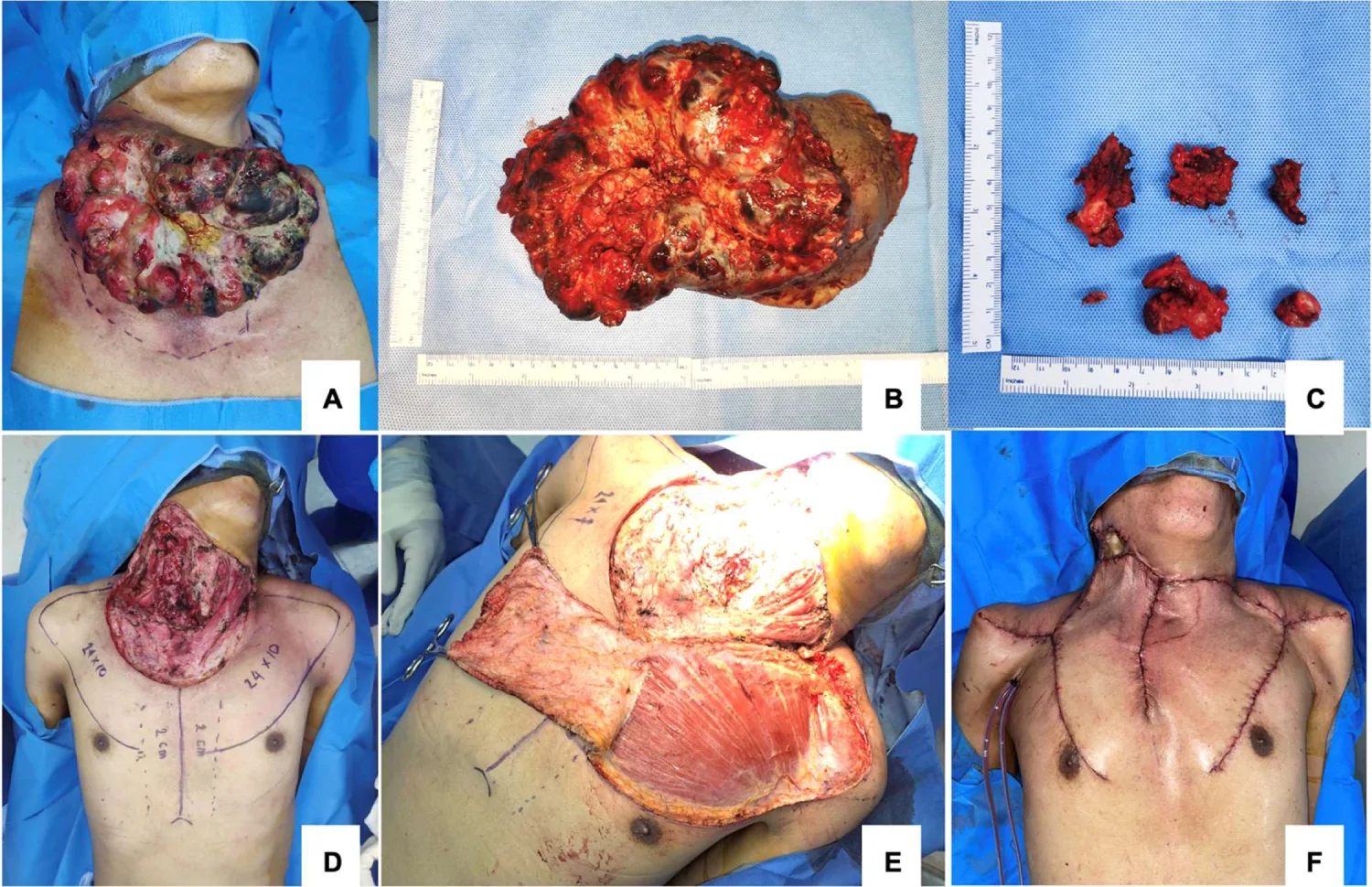
Intraoperative process of debulking and total thyroidectomy with reconstructive surgery using a double-deltopectoral flap: (A) initial presentation; (B) resected mass measuring 12 × 19 × 4 cm; (C) lymph node and benign isthmus nodule; (D) post-tumor debulking and double-deltopectoral flap marking; (E) process of wound closure; (F) postoperative results.

Postoperative histopathology showed a solid-trabecular tumor with oncocytic features and necrosis. Immunohistochemistry (IHC) showed AE1/3 and TTF-1 positivity, partial expression of CK7 and CD10, weak, focal PAX-8 expression, and negativity for synaptophysin and chromogranin.

The IHC staining pattern supports the diagnosis of DHGTC, with an oncocytic carcinoma component (Supplemental Digital Content Figure 1, available at: https://links.lww.com/IJSCR/A171). One dissected lymph node contained metastatic deposits. Histopathology of the contralateral (left) thyroid lobe revealed a separate, synchronous follicular variant of PTC. Given carotid and tracheal infiltration precluding complete resection, gross residual tumor was intentionally left *in situ* (R2 resection). Final pathologic staging was T4bN1M0.

Wound dehiscence was found 1 month after surgery in the left and right shoulders, but both were successfully closed on an outpatient basis. Complete wound closure was achieved after 3 months. The patient also reports slight pain and stiffness in the left side of the neck and a hoarse voice. Whole-body scan (WBS) showed pathological uptake in the bilateral thyroid beds, with no sign of metastases; thus, ablation therapy is scheduled for the patient (Supplemental Digital Content Figure 2, available at: https://links.lww.com/IJSCR/A171).

### Follow-up

The patient underwent two radioactive iodine therapy (RAI) courses within 5 months after surgery, guided by pre-therapy Tg and TSH levels, receiving a cumulative dose of 130 mCi. Serum Tg declined from 21.96 to 7.57 ng/mL between courses. Post-therapy whole-body I-131 scintigraphy demonstrated persistent RAI-avid residual thyroid tissue in the bilateral thyroid beds (predominantly right-sided) without evidence of locoregional or distant metastasis. Follow-up contrast-enhanced neck CT showed a reduction in tumor dimensions from 11.6 × 13.8 × 10.4 cm to 7.5 × 8.7 × 5.4 cm (>30% reduction), consistent with a partial response per respon evaluation criteria in solid tumors 1.1 (RECIST 1.1); cervical lymphadenopathy was reactive rather than malignant. Per the 2015 American Thyroid Association (ATA) response-to-therapy criteria, the patient’s disease status is best classified as a structural incomplete response. No further RAI or systemic therapy has been administered since the second course; management has consisted of close surveillance with serial Tg/TSH monitoring and follow-up imaging. Should structural or biochemical progression emerge, escalation to systemic therapy (e.g., tyrosine kinase inhibitors) would be considered in a multidisciplinary setting.

### Chronological timeline

The chronology of the patient’s timeline is provided in Table 1.

**Table 1:** Chronological timeline of clinical course.

Time	Clinical events
3 years before admission	Patient first noticed a small (quail-egg sized) right anterior neck mass
Following months	The mass progressively enlarged despite herbal treatment and was accompanied by neck pain and dysphagia
1 month before admission	Tumor ulcerated with necrosis, purulent discharge, and recurrent spontaneous bleeding. Progressive anemia developed requiring repeated blood transfusions
Day 0 (admission)	Presented with a 15 × 13 × 10 cm bleeding neck mass. Hemoglobin level was <4 g/dk. Hemostatic agents and epinephrine failed to control bleeding. Initial investigations include laboratory tests, core needle biopsy, contrast-enhanced CT and CT angiography demonstrating a hypervascular thyroid tumor with carotid and internal jugular vein involvement
Days 1–3	Emergency selective embolization of multiple feeding branches from the right external carotid artery using polyvinyl alcohol (PVA) particles achieved temporary hemorrhage control
3 weeks later	Persistent bleeding prompted a second endovascular intervention with placement of a 6 × 58 mm BeGraft covered stent between right common carotid artery and right internal carotid artery
Post intervention	Debulking surgery followed by total thyroidectomy with resection of the infiltrated right internal jugular vein and strap muscles, preservation of the recurrent laryngeal nerve, tracheal shaving and reconstruction using a double deltopectoral flap with infraumbilical full-thickness skin graft
Post operative pathology	Histopathology and immunohistochemistry confirmed differentiated high-grade thyroid carcinoma (DHGTC) with oncocytic feature
1 month after surgery	Bilateral shoulder wound dehiscence occurred and was successfully managed with outpatient wound closure
3 months after surgery	Complete wound healing was achieved. Mild hoarseness and neck stiffness persisted. Whole-body scan demonstrated residual uptake in the thyroid bed without distant metastasis and radioactive iodine therapy was planned

### Methodology

The work has been reported in line with the SCARE criteria^[^6^]^.

## Discussion

The recent WHO 2022 classification added “High-grade follicular cell-derived carcinoma,” which consists of poorly differentiated thyroid carcinoma (PDTC) and DHGTC. PDTC is defined by a solid, trabecular, or insular growth pattern and high-grade features such as increased mitotic index (≥3 per 2 mm^2^) and necrosis, representing a lineage intermediate between well-differentiated and anaplastic carcinoma. In contrast, DHGTC retains the architectural and nuclear features of differentiated carcinoma (e.g., papillary, follicular, or oncocytic morphology) but demonstrates high-grade characteristics such as a mitotic count of ≥5 per 2 mm^2^ and/or tumor necrosis without dedifferentiation into PDTC architecture. This distinction reflects differences in morphology while acknowledging overlapping clinical aggressiveness^[^2,3,7^]^. Currently, no standardized treatment pathways are warranted for DHGTC^[^2,8^]^.

Locally advanced thyroid cancer (LATC) refers to thyroid cancer with gross extension beyond the capsule, invading surrounding structures such as the RLN, trachea, larynx, esophagus, and major neck vessels^[^9^]^. Symptoms include a large neck mass, pain, stiffness, hoarseness, cough, airway obstruction, and dysphagia. LATC also carries a higher risk of distant metastasis and requires thorough evaluation^[^10^]^.

Gross extrathyroidal invasion into surrounding muscle and major vascular structures, as seen in this case, meets established criteria for locally advanced differentiated thyroid cancer, according to current evidence-based surgical guidelines. Imaging and intraoperative findings demonstrated infiltration of the sternocleidomastoid and strap muscles, encasement of the carotid artery, invasion of the IJV, tracheal involvement (SHIN II), and cutaneous ulceration. According to current definitions, invasion of major vessels or soft tissue beyond the thyroid capsule classifies the disease as locally advanced and is associated with increased surgical complexity^[^9^]^.

LATC is often characterized by bulky, locoregional, viscerally invasive disease or rapid progression, both of which require a multidisciplinary approach^[^11^]^. Preoperative evaluation using ultrasonography, contrast-enhanced CT scan, laryngoscopy, or esophagoscopy might be necessary, especially when there are signs and symptoms of involvement of associated structures, to ultimately achieve the goal of complete resection of the mass and preservation of vital structures^[^9^]^. Resection of the strap muscles to achieve a negative margin does not result in significant morbidity. Preoperative vocal cord paralysis is a sign of RLN damage and is a valid indication to sacrifice the RLN^[^10^]^. Attempts to preserve the RLN should be undertaken only if it is possible to achieve removal of all gross tumor. The RLN was preserved in our case.

Airway involvement can be evaluated using SHIN I to IV or McCaffrey I to V staging classifications. The goal for laryngotracheal invasion is complete surgical removal, which can be done via the shave procedure, window resection, circumferential tracheal resection, partial laryngectomy, or total laryngectomy, depending on the extent of the invasion^[^12^]^. Our case showed minimal invasion of the tracheal cartilage, classified as SHIN II, which can be treated with shave resection. This method only removes the gross tumor without resecting the trachea and has been shown to have lower morbidity as well as a comparable rate to extended resection.

The DHGTC in our case showed extensive vascular involvement. In alignment with previous literature, our case also showed IJV infiltration via CT angiography; thus, resection was performed. Resection of a single IJV does not cause major morbidity. For carotid artery involvement, bilateral major veins involvement, or thrombus in a major vein, consultation with a vascular surgeon should be considered^[^9^]^. The hypervascular nature of the case caused the patient to experience multiple episodes of bleeding; thus, multiple vascular interventions consisting of embolization and BeGraft placement of the feeding artery were our novel approach to the case.

Preoperative embolization is a relatively new approach in advanced thyroid carcinoma. It involves intentional vessel occlusion to stop bleeding or reduce tumor growth. While widely used in hepatocellular carcinoma, renal carcinoma, bone metastases, and palliative oncology settings, its role in thyroid cancer – particularly preoperatively – remains uncommon^[^8^]^. Preoperative embolization in highly vascular head and neck tumors has been shown to reduce intraoperative blood loss, and while evidence in thyroid malignancy is limited, existing case series suggest it may facilitate safer surgical resection^[^13^]^. Previous reports (Tables 2–3) showed reduced intraoperative bleeding and occasional tumor shrinkage. Stent graft placement was selected in this case due to persistent hemorrhage and carotid artery encasement, in which vessel sacrifice posed an unacceptable risk of cerebral ischemia. Previous studies have demonstrated that stent grafting can provide immediate hemostasis and maintain cerebral perfusion^[^14^]^. Postembolization fever, as in our case, is a common transient effect. Supportive therapy with antibiotics, corticosteroids, nonsteroidal anti-inflammatory drug, and antithyroid drugs is often required. Surgery is usually performed days to weeks later, depending on the embolization material used.

**Table 2:** Case reports utilizing preoperative embolization on thyroid nodules.

Characteristic	Diagnosis	Vascular embolized	Method of embolization	Duration between embolization and surgical	Compilation postembolization	Medication postembolization	Surgical outcome	Source
29 Y.O. male	Large PTC with extension to the mediastinum and compression of the trachea	Bilateral superior thyroid arteries and left inferior thyroid artery	Gelatin sponge particles	4 days	Mild anterior neck pain mild fever	-	No need for blood transfusion	^[^14^]^
51 Y.O. women	Large PTC-goiter with extension to the mediastinum and compression of the trachea	Bilateral superior, and inferior artery	PVA 150–300 mm	7 days	-	Cephradine (2 g intravenously, twice a day) and prednisone (15 mg daily) for up to 7 days.	Reduction in tumor size reduction in expected blood loss	^[^15^]^
61 Y.O. male	Large cervico- mediastinal multinodular hyperfunctioning goiter with COPD	Superior and inferior left, and inferior right thyroid arteries	Contour emboli 150 mm	30 days	Mild anterior neck pain severe leukocytosis increase in thyroid hormone	Antibiotic prophylaxis with amoxicillin and clavulanate (875 mg þ 125 mg, two times a day), NSAID, prednisone 3 × 5 mg/day, methimazole 3 × 5 mg/day	Reduction in tumor size reduction in expected blood loss	^[^16^]^
71 Y.O. male	Hypervascular metastatic left clavicle lesion of the thyroid	Transverse cervical, suprascapular, clavicular and acromial branches of the thoracoacromial arteries	Gelatin sponge particles	1 day	-	-	Reduction in expected blood loss	^[^17^]^
							Easy separation	
							Necrosis of tumor	
64 Y.O. woman	Hypervascular metastatic sternum lesion of the thyroid	Internal mammary arteries bilaterally	Gelatin sponge particles	Same day	-	-	Complete devascularization and necrosis of the sternal metastasis	^[^17^]^

COPD, chronic obstructive pulmonary disease; NSAID, nonsteroidal anti-inflammatory drugs

**Table 3:** Clinical studies utilizing preoperative embolization of thyroid arteries.

Study participants	Vascular embolized	Method of embolization	Duration between embolization and surgical	Compilation postembolization	Surgical outcome	Source
13 invasive DTC and 7 advanced stages of inoperable ATC cases	Superior and/or one of the two inferior thyroid arteries	PVA 140–750 mm	Not stated	Hematoma, fever, neck pain + massive increase of Tg in DTC that started after 36–48 hours and did not occur in cases of ATC.	In DTC showed decrease operating time, blood loss, and drainage	^[^18^]^
10 large toxic goiters of second degree	Superior and/or one of the two inferior thyroid arteries	PVA 140–750 mm	Within 36 hours	Hematoma, fever, neck pain + a massive increase of thyroglobulin concentrations and a moderate increase of free thyroid hormones, together with a fall of TSH concentration after 48 hours	Significantly shortened the operating time and reduced drainage and blood loss during thyroidectomy	^[^19^]^
22 patients with Graves’ disease	Bilateral superior and unilateral inferior arteries	PVA 150–300 mm	2–3 weeks after on 6 patients	Fever within the first week, neck pain increases in FT3 & FT4 in the first week then declines to normal level the following 2–8 weeks	Reduction in thyroid size (30%–50% of the original size)	^[^20^]^

ATC : advanced thyroid cancer; DTC : differentiated thyroid carcinoma; FT3 : free triiodothyronine; FT4 : free thyroxine.

Careful assessment of intracranial blood flow is essential when performing preoperative embolization or stent graft placement involving the carotid circulation. In this case, CT angiography confirmed preserved cerebral perfusion, and no neurological or ischemic complications occurred. Selective embolization for internal carotid artery occlusion is critical to minimizing stroke risk and ensuring procedural safety.

Outside its use in thyroid carcinoma, embolization of the thyroid arteries can be used in the treatment of goiter, as shown in a clinical study involving 56 patients with goiter, who showed a decrease in goiter size and an improvement in thyroid hormones^[^15^]^. A transient hormone surge postembolization is common, particularly in differentiated lesions capable of hormone storage. In our patient, bleeding initially subsided but recurred mildly within a week. Three weeks later, BeGraft placement in the E-RCA feeder stabilized bleeding, enabling surgery the following day.

Lenvatinib was considered for use as neoadjuvant chemotherapy for this patient in the hope of making the tumor more resectable, in line with the American Head and Neck Society^[^2^]^. However, it was not administered in this case due to the high risk of fatal hemorrhage from its anti-vascular endothelial growth factor activity, although case reports show that lower doses may reduce tumor size and detachment of the tumor from the IJV^[^16^]^.

RAI therapy, guided by clinicopathologic factors, Tg and TSH levels, and lesion uptake, is recommended by the National Comprehensive Cancer Network for remnant ablation or adjuvant treatment^[^7^]^. Unlike well-differentiated thyroid malignancies that usually retain RAI avidity, DHGTCs often show reduced avidity^[^21^]^. A study showed that 47.3% were non-avid, which correlated with lower-grade histology, less vascular invasion, and higher nodal metastasis; however, survival outcomes were not significantly affected by RAI avidity^[^7^]^. Since Tg is produced at lower levels in DHGTCs, the ATA recommends fluorodeoxyglucose - positron emission tomography for surveillance^[^2^]^. RAI therapy was still given in this case because WBS showed uptake in the thyroid bed.

This case report presents a rare and complex case of DHGTC with severe anemia, successfully managed through a staged approach of embolization, stent grafting, and surgery. A key strength of this case report lies in the multidisciplinary vascular–oncologic approach, which enabled staged hemorrhage control through embolization and stent graft placement prior to definitive surgical resection, supported by detailed radiologic and intraoperative documentation. Nevertheless, several limitations must be acknowledged: the rarity of DHGTC inherently restricts the generalizability of our findings, and standardized guidelines for preoperative vascular intervention in thyroid carcinoma are currently lacking. In addition, long-term oncologic and functional outcomes remain to be determined. Despite these limitations, this case contributes meaningful clinical insight into the management of hemorrhagic, hypervascular, advanced thyroid malignancies.

## Conclusion

This case exemplifies the complexity and challenges of managing locally advanced DHGTC. The tumor’s aggressive nature, extensive vascular involvement, and significant local invasion highlight the necessity for a multidisciplinary approach. Our case showed extensive bleeding from the mass that was treated with preoperative embolization and BeGraft placement. This vascular intervention enabled surgical debulking and total thyroidectomy with reconstructive surgery in the patient, with lower intraoperative bleeding. Preoperative embolization of the thyroid arteries and placement of BeGraft stents in our case were beneficial in the management of bleeding in large, hypervascular thyroid cancer.

## Data Availability

Additional data can be requested if needed.
